# Supplementary material for: Comprehensive analysis of bioplastics: life cycle assessment, waste management, biodiversity impact, and sustainable mitigation strategies
Source: PeerJ. 2024 Sep 11;12:e18013. doi: 10.7717/peerj.18013 (PMC11401513; doi:10.7717/peerj.18013)
Supplement: Supplemental Information 1 — The life cycle of bioplastics, from the biorefining of non-edible feedstock and oil to monomer production, polymerization, and formulation into bioplastic products. It also depicts various degradation pathways, including physical (incineration), chemical (thermolysis and solvolysis), and biological (aerobic, anaerobic, and genetic modification) methods, as well as recycling options (Ali et al., 2023). [file peerj-12-18013-s001.docx]

**Supplementary Figure**


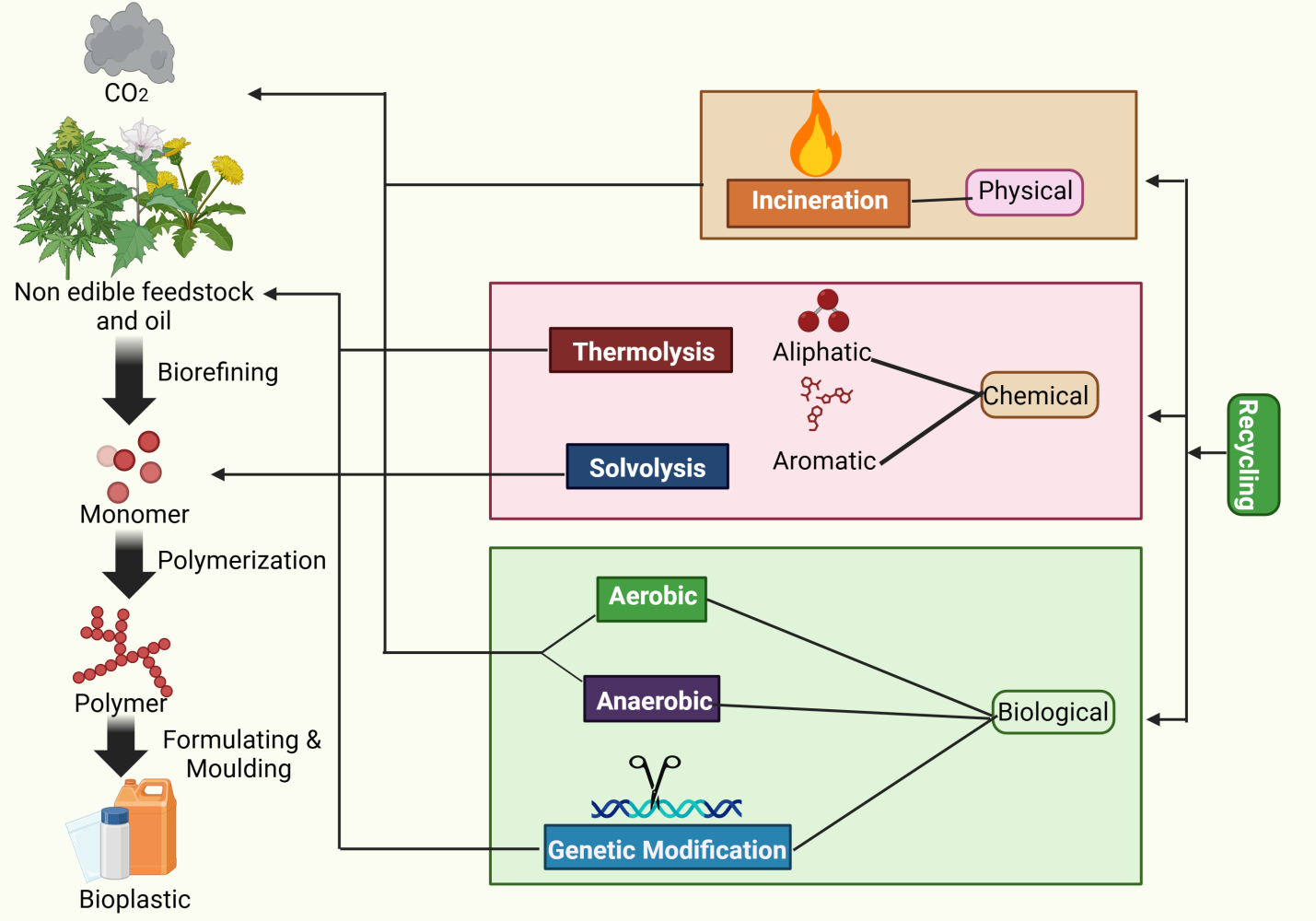


**Figure S1: Comprehensive Lifecycle and Degradation Pathways of Bioplastics.** This figure outlines the lifecycle of bioplastics, from the biorefining of non-edible feedstock and oil to monomer production, polymerization, and formulation into bioplastic products. It also depicts various degradation pathways, including physical (incineration), chemical (thermolysis and solvolysis), and biological (aerobic, anaerobic, and genetic modification) methods, as well as recycling options (Modified from Ali et al., 2023, https://www.sciencedirect.com/science/article/pii/S2666498423000194).
